# Subject: evaluation of mandibular bone abnormalities in CKD patients using CBCT

**DOI:** 10.2340/aos.v85.45207

**Published:** 2026-01-09

**Authors:** Suhani Ghai, Ankit Grover, Kailash N. Singh

**Affiliations:** a Oral and Maxillofacial Surgery, Prasanthi Clinic, New Delhi, India; b Department of Nephrology, Indraprastha Apollo Hospital, New Delhi, India

Dear Editor,

We read with great interest the article titled *‘Evaluation of mandibular bone abnormalities in patients with chronic kidney disease using cone beam computed tomography’* [1]. This retrospective study provides valuable insights into the association between mandibular bone anomalies and chronic kidney disease, and the authors deserve commendation. Their work expands the limited literature on cone beam computed tomography (CBCT)-based morphometric indices in medically compromised patients and underscores the link between bone quality and systemic biochemical markers.

However, while we commend the novelty and relevance of this work, we would like to raise certain concerns that may limit the generalizability and clinical applicability of the findings. Firstly, the study excluded the maxilla and concentrated mostly on evaluating the mandibular bones. Omitting the maxilla limits the results’ relevance for thorough pre-implant planning because of its unique anatomical and biological characteristics as well as its crucial function in implant dentistry. Secondly, although diabetes mellitus was considered, other important confounders such as smoking status, dialysis duration and modality, and the use of medications including corticosteroids, phosphate binders, and vitamin D analogs were not fully accounted for in the analysis. By restoring normal mineral metabolism and averting renal osteodystrophy and bone fractures linked to elevated phosphate levels, phosphate binders enhance bone quality in patients with chronic kidney disease (CKD) [2]. Similarly, vitamin D analogs can treat mineral imbalances and reduce parathyroid hormone (PTH) (secondary hyperparathyroidism), which directly hinders bone production, to enhance bone quality in those with CKD [3]. These factors could independently influence bone metabolism and may have affected the outcomes.

Thirdly, the study’s external validity may be limited to other ethnic and geographic groups with different CKD profiles because it only included Korean patients from one tertiary centre. Lastly, while CBCT morphometric changes were meticulously recorded, the study did not directly connect these results to real implant outcomes such as osseointegration or long-term survival, which would have improved its clinically applicable relevance.

In summary, this study makes a significant contribution to the growing body of knowledge regarding CKD-related skeletal changes. At the same time, further prospective, multicentre investigations – including both maxillary and mandibular bones, a broader set of confounders, and longitudinal implant outcome data – are warranted to confirm these findings and strengthen their clinical utility.
